# Fabrication of device with poly(*N*-isopropylacrylamide)-*b*-ssDNA copolymer brush for resistivity study

**DOI:** 10.1186/s12951-017-0303-4

**Published:** 2017-10-05

**Authors:** Yi-Zu Liu, May-Show Chen, Chih-Chia Cheng, Shih-Hsun Chen, Jem-Kun Chen

**Affiliations:** 1 Department of Materials Science and Engineering, National Taiwan University of Science and Technology, 43, Sec 4, Keelung Rd, 106 Taipei, Taiwan, ROC; 2 School of Oral Hygiene, College of Oral Medicine, Taipei Medical University, Taipei, 11031 Taiwan, ROC; 3 Department of Dentistry, Taipei Medical University Hospital, Taipei, 11031 Taiwan, ROC; 4 Graduate Institute of Applied Science and Technology, National Taiwan University of Science and Technology, 10607 Taipei, Taiwan, ROC; 5 Department of Mechanical Engineering, National Taiwan University of Science and Technology, 43, Sec 4, Keelung Rd, 106 Taipei, Taiwan, ROC

**Keywords:** Block copolymer brush, Supramolecular complex, Resistivity

## Abstract

In this study, we grafted bromo-terminated poly(*N*-isopropylacrylamide) (PNIPAAm) brushes onto thin gold films deposited on silicon, and then reacted with NaN_3_ to produce azido-terminated PNIPAAm brushes. A probe sequence of single-stranded DNA (ssDNA) with a 4-pentynoic acid succinimidyl ester unit was grafted onto the azido-terminated PNIPAAm brushes through a click reaction, resulting in the formation of block copolymer brushes. The PNIPAAm-*b*-ssDNA copolymer brushes formed supramolecular complexes stabilized by bio-multiple hydrogen bonds (BMHBs), which enhanced the proton transfer and thereby decreased the resistivity of the structures. In addition, the optimal operation window for DNA detection ranges from 0 to 0.2 M of NaCl concentration. Therefore, the specimens were prepared in the PBS solution at 150 mM NaCl concentration for target hybridization. The supramolecular complex state of the PNIPAAm-*b*-ssDNA copolymer brushes transformed into the phase-separated state after the hybridization with 0.5 ng/µL of its target DNA sequence owing to the competition between BMHBs and complementary hydrogen bonds. This phase transformation of the PNIPAAm and probe segments inhibited the proton transfer and significantly increased the resistivity at 25 °C. Moreover, there were no significant changes in the resistivity of the copolymer brushes after hybridization with the target sequence at 45 °C. These results indicated that the phase-separated state of the PNIPAAm-*b*-ssDNA copolymer brushes, which was generally occurred above the LCST, can be substantially generated after hybridization with its target DNA sequence. By performing the controlled experiments, in the same manner, using another sequence with lengths similar to that of the target sequence without complementarity. In addition, the sequences featuring various degrees of complementarity were exploited to verify the phase separation behavior inside the PNIPAAm-*b*-ssDNA copolymer thin film.

**Electronic supplementary material:**

The online version of this article (doi:10.1186/s12951-017-0303-4) contains supplementary material, which is available to authorized users.

## Background

DNA is an interesting molecular material that shows a long rod-like duplex structure with base-pair (bp) stacking.  The base separation and the diameter of the duplex were 3.4 and 20 Å respectively. It, therefore, appears to be a good candidate for the one-dimensional energy transfer and conduction along with π-electron clouds of stacked bases. However, the quantity of DNA in the specimen is usually small, so a medium material to associate with DNA is quite necessary. Macroscopic electric conductivity has been studied by using fibrous gels in which duplex DNAs are randomly oriented [1]. Photoinduced electron transfer between redox dye molecules intercalated in the DNA molecules has been studied in homogeneous aqueous solution [2]. These reports encouraged us to prepare a complex incorporating with DNA as a conductive film of the supermolecular amphiphile, in which receptors were interacted with targets to vary the conductivity [3–5]. One of the most highly characterized thermosensitive amphiphiles is poly(*N*-isopropylacrylamide) (PNIPAAm), which contains hydrophilic amido groups and hydrophobic isopropyl groups [6, 7]. PNIPAAm is soluble in water up to its lower critical solution temperature (LCST), which was approximately 31–32 °C [8], because of amido groups forms hydrogen bonds (HB) with the surrounding water molecules [9, 10]. Integration DNA with polymers can modulate the properties of biological entities, forming new hybrid materials well known as self-assembled supramolecules under thermal or pH stimulus [11, 12]. Hydrogen bonding (HB) has been used as a general design element in many supramolecular polymer assemblies [13–15]. The incorporation of nucleobases into the synthetic polymers enables their supramolecular assembly, which may be applied in molecular electronic devices.

We have found that bio-multiple hydrogen bonds (BMHBs) between PNIPAAm and adenine (A) can be exploited to change the morphology, crystalline structure, and temperature-responsiveness of PNIPAAm merely by varying the concentration of A [16]. The presence of BMHBs can enhances the degree of electron transport in a PNIPAAm film, leading to remarkable changes in the electrical conductivity. The initially insulating PNIPAAm can be transformed into its semiconducting form through this simple process [11]. In this study, we formed the novel DNA recognition layers with PNIPAAm-*b*-ssDNA copolymer brushes as aptamers, which changed conductivity upon complexation and phase separation of the PNIPAAm and ssDNA segments. Our target sequence was the DNA strand complementary to the ssDNA segment of the copolymer brushes. When we added the target molecule, its binding to the ssDNA component led to phase separation of the PNIPAAm and ssDNA segments. Thus, the conductivity changed because of HB between the target sequence and ssDNA was much stronger than the BMHBs between the PNIPAAm and ssDNA segments. We characterized the supramolecular complexation/decomplexation of the PNIPAM-*b*-ssDNA copolymer brushes with resistivity.

## Experimental section

### Materials

Silicon wafers, Si(100), polished on one side (diameter: 6 in.) were supplied by Hitachi (Japan) and cut into 0.6 × 0.6 in. samples. *N*-Isopropyl acrylamide (NIPAAm, Acros Organics) was recrystallized from toluene/hexane (50%, v/v) and dried under a vacuum prior to use. The other materials used for graft polymerization were purchased from Acros Organics such as 3-aminopropanethiol (AT), 2-bromo-2-methylpropionyl bromide (BIBB), copper(I) bromide, copper(II) bromide, triethylamine (TA), 1,1,4,7,7-pentamethyldiethylenetriamin (PMDETA), and sodium azide (NaN_3_). PMDETA, AT, and BIBB were purified through vacuum distillation prior to its use. All other chemicals and solvents were of reagent grade and purchased from Aldrich Chemical. The target sequence was 5′-GACTT-GCCAT-CGTAG-AACTG-3′ and its complementary probe sequence was NH_2_-(CH_2_)_6_-5′-CAGTT-CTACG-ATGGC-AAGTC-3′. The control sequences were 5′-AGTCG-TGTAC-AGTTG-TGACT-3′ (CS0), 5′-AGTCG-TGTAC-AGTTG-AACTG-3′(CS25), 5′-AGTCG-TGTAC-CGTAG-AACTG-3′(CS50), and 5′-AGTCG-GCCAT-CGTAG-AACTG-3′(CS75) featuring 0, 25, 50 and 75% of complementarities, respectively. 20 base pairs in the target could be considered as the combination of four segments and each segment included the five base pairs. The segments with underline represented the matched segments with the probe sequence. The complementarities were defined as the ratio of the amount of matched segments to the total four of segments. The target, CS75, CS50 and CS25 oligomers were purchased from TriLink Biotechnologies (HPLC-purified for highest purity; San Diego, CA) [17–19]. 4-Pentynoic acid succinimidyl ester was synthesized according to the literature [20].

### PNIPAAm-*b*-ssDNA copolymer brushes

**Fig. 1:**
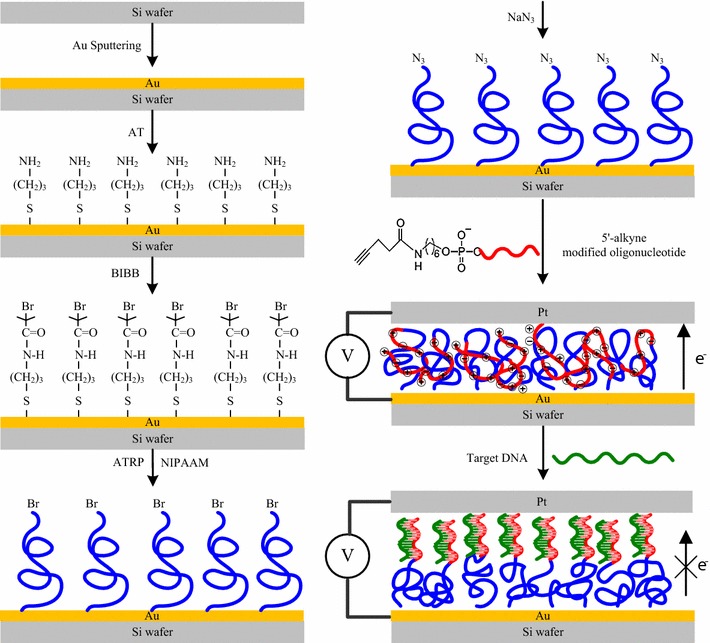
Schematic representation of the process used to graft the PNIPAAm-*b*-DNA copolymer brushes

The length of the polymer was varied to tune the BMHBs between the PNIPAAm and ssDNA segments in the PNIPAAm-*b*-ssDNA copolymer brushes. Figure 1 outlines the synthetic pathway for anchoring the initiators on the sputtered gold thin film of a silicon substrate, which acts as the bottom electrode. An NH_2_-terminated self-assembled monolayer (SAM) was first fabricated and then reacted with BIBB to obtain initiators for an atom-transfer radical polymerization (ATRP). To immobilize the ATRP initiator, the as-prepared substrate was immersed in 0.5% (w/v) solution of AT in toluene for 2 h at 50 °C. The AT units were assembled on the surface of Au through their thiol groups. The sample was immersed in 2% (v/v) solution of both BIBB and TA in toluene for 8 h at 20 °C. As the next step, as-prepared samples were placed in a Soxhlet apparatus to remove any non-grafted material. The surface-initiated ATRP grafting of NIPAAm was performed in a glass vessel purged with N_2_. After that NIPAAm, PMDETA, Cu(I)Br, and CuBr_2_ were dissolved in dimethylformamide (DMF) [5, 21]. The reaction solution was sonicated for 2 min and then added to a glass vessel in which the AT/BIBB-functionalized wafers had been placed. Polymerization was performed at room temperature under N_2_ purging for 4, 8, 12, 16, or 20 h. After the desired period of time, the obtained bromo-PNIPAAm—grafted samples were removed from the solution, rinsed with the copious amounts of deionized water to remove any unreacted monomer and non-grafted PNIPAAm, and then dried under the flow of N_2_. Azido-PNIPAAm brushes were obtained on the surfaces after treating the bromo-PNIPAAm brushes with NaN_3_. The substitution reaction was carried out for overnight by exposing the bromo-terminated substrates to a saturated solution of NaN_3_ in DMF in a covered container. The sample was then rinsed with DMF followed by methanol and DI water before drying in the stream of N_2_. After these reactions, the wafers were placed in a Soxhlet apparatus to remove any non-grafted materials and then dried under N_2_ prior to use. A solution of the 4-pentynoic acid succinimidyl ester-conjugated probe sequence (100 nmol) in phosphate buffer (PB, pH 7.2; 20 mM, 2.5 mL) was added to purged glass vessel containing a grafted azido-PNIPAAm sample and DMF (2.5 mL) to perform the click reaction. In addition, ssDNA was also grafted onto an azido-modified Au substrate without PNIPAAm in a blank experiment to analyze the thickness. A stock solution (0.1 mL) of catalyst/ligand (1.0 mmol of CuSO_4_/1.1 mmol of tris[(1-benzyl-1H-1,2,3-triazol-4-yl)methyl]amine) in dimethyl sulfoxide (DMSO)/water (1:1, v/v) and sodium ascorbate (0.79 mg, 4.0 mmol) was then added. The reaction mixture was kept at 15 °C for 20 h. The as-prepared samples were incubated for overnight at room temperature in the solution and then washed three times with washing buffer [10 mM Tris–HCl (pH 7.5), 150 mM NaCl, 0.05% Tween20] and three times with water. The samples were then dried under N_2_ to ensure that their surfaces were dry and free of dust particles. Herein, the surfaces with PNIPAAm grafted for 4, 8, 12, 16 and 20 h and sequentially grafted with the ssDNA to form the aptamers of the PNIPAAm-*b*-ssDNA copolymer brushes were denoted as PN4D, PN8D, PN12D, PN16D, and PN20D respectively. The target sequence was diluted to the final concentration of 50 μM with a hybridization buffer comprising SSC (150 mM NaCl, 0.3 M sodium citrate·2H_2_O, pH 7), Denhardt’s solution (1% bovine serum albumin), 2% Ficoll400, 2% polyvinylpyrrolidone, and 0.5% sodium dodecyl sulfate. The two small strips of adhesive tape were affixed along the rim of the substrate and then covered with a clean microscope slide cover glass. The cavity that formed between the chip and the microscope slide cover glass was filled by slow loading of hybridization solution (ca. 30 μL) under capillary force. After that the cover glass was removed, the chip was washed three times with washing buffer and then with the copious amounts of water and dried under N_2_. Further, experiments were performed in the same controlled manner using CS0, CS25, CS50 and CS75, which had lengths similar to that of the target sequence with various complementarities. The water contact angles of the PNIPAAm-*b*-ssDNA copolymer brushes were measured after they had been dried under the flow of N_2_. The samples were immersed in the water at 25 and 45 °C for 3 h to observe the phase separation behavior, respectively. To remove the residual H_2_O molecules promptly, the samples were lyophilized by a commercial freeze dryer (EYELA FDU-1200) under 1.5 mTorr prior to further analysis. The chemical compositions of the modified silicon surfaces were determined through photoelectron spectrometry (XPS, Scientific Theta Probe, UK) [22]. The thicknesses of the copolymer grafts on the silicon substrates were measured by using ellipsometry (SOPRA SE-5, France). The thickness and surface chemical composition of the copolymer in water should be remain nearly at 25 and 45 °C after lyophilization.

### Assessment of BMHBs through effective equilibrium constant of layers (*K*_e_)

HB between the PNIPAAm and probe segments at temperatures below and above the LCST led to respective complexation and decomplexation resulted in changes to the thicknesses of the copolymer brushes and thermoresponsive behavior of the surfaces. The length of the probe segment was kept constant while varying the length of the PNIPAAm. The samples were subjected to complex/decomplex in a water bath at a given temperature until an equilibrium state had been established. The Variations in the BMHBs of PNIPAAm-*b*-ssDNA copolymers were monitored by using ellipsometry. As the thicknesses of the PNIPAAm and ssDNA segments within a copolymer were related directly to their concentration, it was possible to obtain the values of effective equilibrium constant of layers (*K*_e_) (1/nm) of the PNIPAAm-*b*-ssDNA copolymers from a linearized Benesi–Hildebrand equation, allowing quantitative characterization of the interactions between the PNIPAAm and the ssDNA at temperatures below and above the LCST [16]. For the binary PNIPAAm-*b*-ssDNA complexation, the following equilibrium was considered:

1 $$\begin{aligned} & {\text{PNIPAAm-}}b{\text{-ssDNA (complexation)}}\\ & \quad \Leftrightarrow {\text{ PNIPAAm-}}b{\text{-ssDNA (separation)}} \end{aligned}$$

and the effective equilibrium constant was given by

2 $$K_{\text{e}} = \frac{{[{\text{PNIPAAm-}}b{\text{-ssDNA segment}}]}}{{[{\text{PNIAAm segment}}][{\text{ssDNA segment}}]}}$$

The Benesi–Hildebrand equation was used to calculate the values of *K*_e_:

3 $$\frac{1}{\Delta H} = \frac{1}{{K_{e} \Delta H_{\text{max} } [ {\text{PNIPAAm}} \,segment ]}} + \frac{1}{{\Delta H_{\text{max} } }}$$

where Δ*H* is the change in the thickness of PNIPAAm-N_3_ after grafting to ssDNA; Δ*H*_max_ is the thickness of the ssDNA segment in the absence of the PNIPAAm segment; and [PNIPAAm segment] represents the thickness of PNIPAAm-N_3_ at temperatures below and above the LCST. The slope of the double-reciprocal plot would be equal to 1/*K*_e_Δ*H*_max_, with an intercept equal to 1/Δ*H*_max_, from which values of *K*_e_ can be obtained.

### Surface properties of the PNIPAAm-*b*-ssDNA copolymer brushes

The static water contact angles (SWCAs) were measured by using a contact angle meter (Sindatek Instruments). The temperature of the glass slides during the SWCA measurements were controlled by placing the slides on the top surface of an aluminum stage, the temperature of which was controlled by a water bath at either 25 °C (below the LCST) or 45 °C (above the LCST) [23]. The surface morphologies of the membranes were investigated through the scanning electron microscopy (SEM) using JEOL JSM 6500F instrument operated at 15 kV.

The electroactivities of the PNIPAAm-*b*-ssDNA copolymer brushes were investigated through the measurement of *I*–*V* characteristics. The PNIPAAm-b-ssDNA was grafted to an Au thin film (bottom electrode). Sequentially, the copolymer-grafted samples were hybridized with target and control sequences successively. After cleaning and lyophilizing processes, Pt electrodes (thickness: 200 nm) were deposited onto the copolymer films as a top electrode by using a stainless-steel shadow mask as the top electrode and the samples were measured for* I*–*V* characteristics from the top to the bottom electrode of the Au substrate. The resistivity of each copolymer film was measured at 25 and 80 °C and the voltage sweeps were recorded from − 0.2 to + 0.2 V [11, 24].

## Results and discussion

### Surface properties of the PNIPAAm-*b*-ssDNA copolymer brushes

**Fig. 2:**
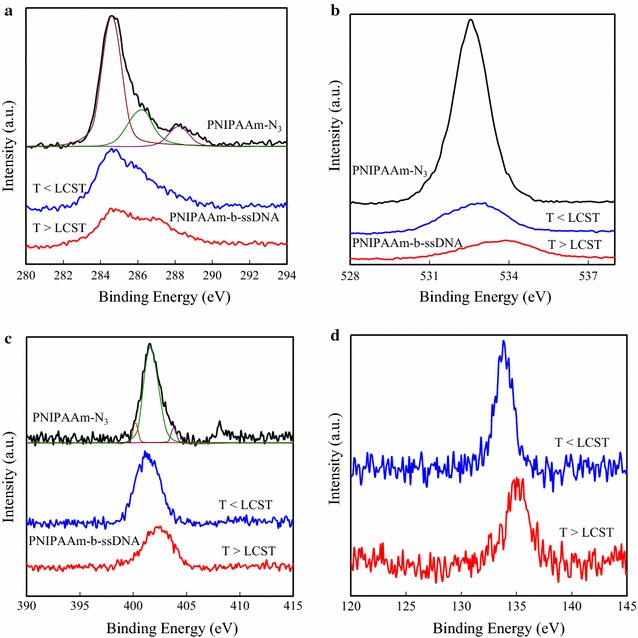
High-resolution **a** C 1s, **b** O 1s, and **c** N 1s core level XPS spectra of PNIPAAm-N_3_ and PNIPAAm-*b*-ssDNA polymer brushes grafted onto Au surfaces for 20 h and **d** P 2p core level XPS spectra of PNIPAAm-*b*-ssDNA polymer brushes, all recorded at temperatures below and above the LCST

XPS was employed to determine the chemical compositions of the Au surfaces at various stages during the surface modification process as well as the presence of the grafted PNIPAAm-*b*-ssDNA copolymer brushes [8]. After polymerization for 20 h, the chemical compositions determined of 427 nm thick layer of PNIPAAm-N_3_ and of PNIPAAm-*b*-ssDNA brushes at temperatures below and above the LCST was shown (Fig. 2). The high-resolution C 1s spectra in Fig. 2a show the value of 12.1:12.9:75.5, for the oxygen: nitrogen: carbon molar ratio for PNIPAAm-N_3_ was consistent with the expected ratio of 12.5:12.5:75.0 for PNIPAAm. The obtained value after curve-fitting using the signals with binding energies (BEs) of 284.6, 286.2, and 288.2 eV, was attributed to the C–C/H, C–N, and C=O chemical bonding environments respectively. The BE for the C=O groups was slightly higher than the previously reported value of Yu et.al [25]. It was also noted that the BE for the C=O groups of PNIPAAm increased slightly after azido-terminating the surface. In addition, the peak area ratios of C–N and C=O to C–C/H were both significantly greater for the PNIPAAm-*b*-ssDNA brush surface, which was consistent with ssDNA grafting [26]. Moreover, the PNIPAAm-*b*-ssDNA surface was heated above the LCST temperature to investigate the HB between its PNIPAAm and ssDNA units. A peak with a BE of 287.1 eV appeared for the PNIPAAm-*b*-ssDNA brush surface above its LCST, presumably representing the BE of the C=O groups of the ssDNA. These results suggested that the BMHBs between PNIPAAm and ssDNA weakened as a result of the increased intramolecular HB of PNIPAAm at temperatures above the LCST. This phenomenon also led to the ssDNA segments being driven up to the surface. The corresponding high-resolution O 1s spectra for PNIPAAm-N_3_ in Fig. 2b shows one peak centered at 532.5 eV in the O 1s region, which is attributable to C=O bonding. At a temperature below the LCST, this peak was shifted to 532.9 eV after grafting of the ssDNA onto the PNIPAAm-N_3_ in the case of above the LCST of the PNIPAAm-b-ssDNA copolymer brushes, the peak shifted further to 533.9 eV. The survey spectrum of the bromo-PNIPAAm brushes featured Br 3d peak and Br 3p peak at 70 and 188 eV respectively along with the other peaks [21]. The bromine atoms were converted into azido groups through substitution with NaN_3_ in DMF. This transformation was clearly evident in the high-resolution N 1s XPS scan (Fig. 2c), with a curve fitted using the peaks with BEs of 400.2, 403.8, and 401.6 eV. The major peak at 401.6 eV was attributed to the CNH units of PNIPAAm. The spectrum features a minor characteristic double peak structure for the azido groups at 400.2 and 403.8 eV, with a BE split of 3.6 eV [27]. The peak areas were in a ratio of 2.6:1, for an azide structure (an internal nitrogen atom surrounded by two nitrogen atoms) as expected. The less-intense peak at 404.2 eV represented the electron-deficient nitrogen atom of the azido groups [28, 29]. The corresponding high-resolution P 2p spectra in Fig. 2d represents the P 2p peak at 133.8 eV was also shifted to 135.2 eV upon heating the PNIPAAm-b-ssDNA copolymer to a temperature above its LCST. Whereas the BE shifts for the O 1s peaks were small (1.0 eV), those of the P 2p peaks were relatively large (1.4 eV). The larger BE shifts have also been observed in the P 2p spectra of pure DNA monolayers, suggesting that the ssDNA segments interacted with the PNIPAAm units through the phosphate atoms in the nucleic acid bases.

**Fig. 3:**
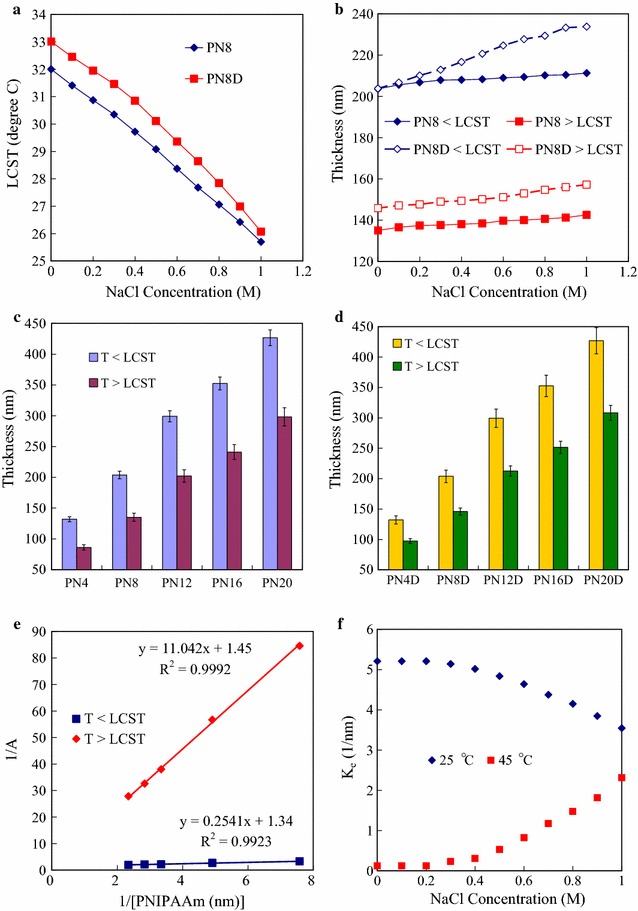
**a** LCSTs, and **b** thicknesses at 25 and 45 °C of PN8 and PN8D measured for NaCl at concentrations from 0 to 1.0 M. **c**, **d** Thicknesses of **c** PN4, PN8, PN12, PN16 and PN20, and **d** PN4D, PN8D, PN12D, PN16D and PN20D copolymer brushes at 25 and 45 °C, respectively. **e** Double-reciprocal plots of 1/*A* with respect to [PNIPAAm segment] at 25 and 45 °C, according to the linearized formula $\frac{1}{\Delta A} = \frac{1}{{K_{e} \Delta A_{{\rm max} } [PNIPAAm \,segment ]}} + \frac{1}{{\Delta A_{{\rm max} } }}$. **f** K_e_ values of PNIPAAm-*b*-ssDNA copolymer brushes plotted as a function of NaCl concentration at 25 and 45 °C, respectively

In general, real specimens are prepared in buffer solutions including cations and anions of salt with concentration ranged from 0.1 to 0.2 M. The salt effect in this system was investigated for further application with real samples. PNIPAAm bears both hydrophilic (amide) and hydrophobic (isopropyl) groups and has been described as a model system for the cold denaturation of peptides and proteins [29]. The salt effects on the LCST of PNIPAAm have been reported and related to the Hofmeister series [30]. All the effects of Hofmeister anions on PNIPAM solvation can be explained by three interactions of the ions with the polymer and its hydration waters. First, the anions can polarize an adjacent water molecule that is in turn involved in hydrogen bonding with the amide. Secondly, these species can interfere with the hydrophobic hydration of the macromolecule by increasing the surface tension of the cavity surrounding the backbone and the isopropyl side chains. Thirdly, the anions may bind directly to the polyamide. The first and second of these effects should lead to the salting-out of the polymers thereby lowering the LCST. The third effect should lead to the salting-in of the polymer [31]. From Fig. 3a shows the initial onset values of the LCST for PNIPAM-N_3_ (PN8) and PNIPAAm-*b*-ssDNA (PN8D) solutions plotted as a function of NaCl concentration, the linear decrease of LCST from 32 to 25.7 °C was observed upon increasing the NaCl concentration to 1 M for homopolymer (PN8). The increasing of NaCl concentrations affect both PNIPAAm and PNIPAAm-*b*-ssDNA solubility in a complex fashion. A higher LCST indicating the coil—globule transition of the PNIPAAm-*b*-ssDNA. Because of the hydrophilic nature of DNA, the LCST of the block copolymer was 2–3 °C higher than those of the homopolymers. (Additional file 1: Figure S1) Therefore, the salt concentration within the copolymers predominately determined the LCST. These results suggested that block copolymer chains with adequate amphiphilicity assemble at the LCST. Figure 3b displays the thicknesses of PNIPAM-N_3_ (PN8) and PNIPAAm-*b*-ssDNA (PN8D) measured for NaCl at concentrations from 0 to 1.0 M. The anions of salt induced the salting-out of the homopolymer resulting the slight increase in the thicknesses of the PN8 layers at both of 25 and 45 °C upon increasing the NaCl concentration. For PN8D, more distinct increase in thickness at 25 °C was attributed to the salt-out of the DNA segment copolymer. The results suggest that the ions within the copolymer facilitate the protonation results in the copolymer layer extension below the LCST. With switching the temperature from 25 to 45 °C, anions binding to the polyamide was accelerated and the collapse of PNIPAAm above the LCST restricting the layer extension. There was significant a difference from the case of the complexed copolymer. This suggests that both decomplexed and complexed copolymer chains are isolated stably without the association between DNA strands at the salt concentration. From Fig. 3c displays the thicknesses of the PNIPAAm-N_3_ layers, which were grown from the Au surfaces via ATRP, after various polymerization times at 25 and 45 °C, there was an approximately linear increases in the thicknesses of the grafted PNIPAAm layers on the Au-AT-BIBB surfaces occurred upon increasing the polymerization time to 20 h, at temperatures both below and above the LCST. The greater thickness of the PNIPAAm brushes grafted onto the Au substrate at temperatures below the LCST provided a so-called brush-like regime of polymer brushes [32, 33]. In contrast, thinner layers were also obtained when the temperature was above the LCST, which was indicative of the formation of globule regimes of the polymer brushes due to chain collapse. Thus, the PNIPAAm brushes formed globule and brush regimes at temperatures above and below the LCST respectively. In addition, the ratio of the thickness in the globule regime to the brush regime was ranged from 61 to 65%. Figure 3d displays the thicknesses of the PNIPAAm-*b*-ssDNA layers at 25 and 45 °C. The thicknesses of the PNIPAAm-*b*-ssDNA layers formed after grafting ssDNA onto PNIPAAm layers of various thicknesses did not vary significantly at 25 °C. This observation suggests that the ssDNA segments were buried within the PNIPAAm segments to form complexes that were stabilized through BMHBs at 25 °C. After grafting ssDNA onto PNIPAAm layers of various thicknesses, significant changes in thickness were evident at 45 °C, which is consistent with phase separation of the two segments. At 45 °C, the intramolecular HB among the PNIPAAm groups was stronger than the BMHBs between the PNIPAAm and ssDNA segments, which caused the ssDNA segments to be driven to the surface. The *K*_e_ values were calculated to quantify the strength of the BMHBs between the PNIPAAm and ssDNA units according to the changes in thicknesses. The plots of Eq. (3) were approximately linear with respect to the increase in the thickness of PNIPAAm segment at both temperatures, indicating that the data fitted the equation well with statistical correlation coefficients (*R* ^2^) of greater than 0.99 (Fig. 3e). The calculated values of *K*_e_ for the PNIPAAm-*b*-ssDNA copolymer brushes at 25 and 45 °C were 5.21 and 0.12 1/nm, respectively. The larger value of *K*_e_ for the PNIPAAm-*b*-ssDNA copolymer brushes at 25 °C suggests an extremely high concentration of complexed PNIPAAm and ssDNA segments with a stronger degree of protonation for their BMHBs. The protonation of the BMHBs weakened significantly at 45 °C, suggesting that the BMHBs between the ssDNA and the PNIPAAm segments was thermodynamically tunable. The ssDNA sequences in the PNIPAAm-*b*-ssDNA copolymer brushes functioned as rich proton donors within the PNIPAAm matrix, suggesting that variable electron transport might be a means of varying the conductivity of such surfaces at temperatures below and above the LCST. Furthermore, the *K*_e_ values for the PNIPAAm-*b*-ssDNA copolymer brushes at 25 and 45 °C, did not change significantly after immersion in NaCl solution below 0.2 M of concentration (Fig. 3f). The values of K_e_ decreased and increased initially upon increasing the NaCl concentration from 0.2 to 1 M at 25 and 45 °C, respectively. The results indicate that residual salts over 0.2 M affect the PNIPAAm solubility in a complex fashion at 25 °C, retarded the complexation of the PNIPAAm segments and ssDNA due to the effect of salting-out of the polymers. However, the anions over 0.2 M binding to the polyamide at 45 °C facilitated the complexation due to the effect of salting-in of the polymers.

**Fig. 4:**
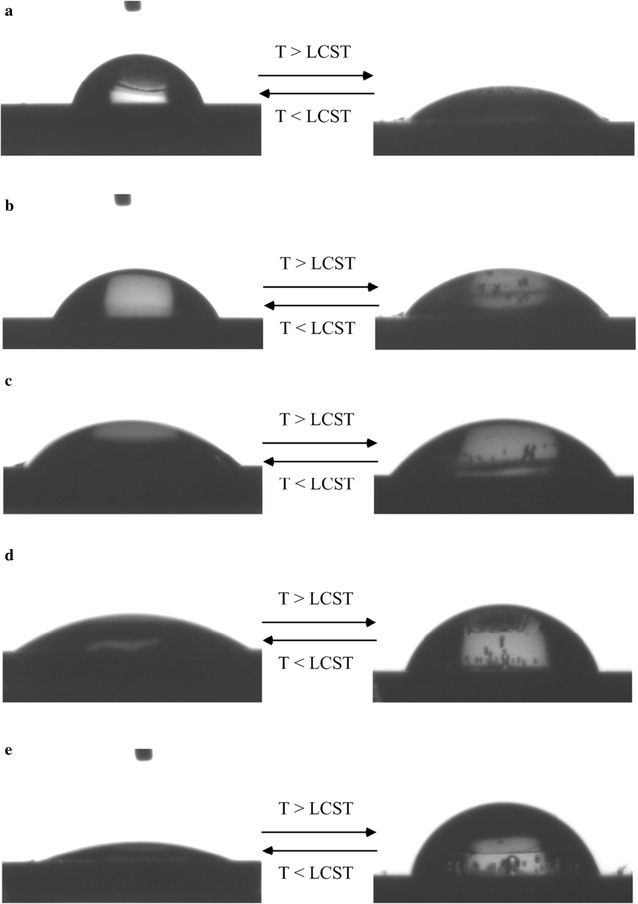
SWCAs of **a** PN4D, **b** PN8D, **c** PN12D, **d** PN16D and **e** PN20D copolymer brushes, measured at 25 and 45 °C

Figure 4 displays the SWCAs of PN4D, PN8D, PN12D, PN16D, and PN20D. Each surface exhibited thermally responsive switching between its hydrophilic and hydrophobic states at temperatures below and above its LCST, respectively. The SWCAs of the PN4D brushes were 78.5 and 46.9° at 25 and 45 °C, respectively (Fig. 4a). This thermal responsivity of PN4D is completely opposite from that of pure PNIPAAm, but can be explained by considering the miscibility of the PNIPAAm and ssDNA segments at 25 °C. The HB interactions between the PNIPAAm and ssDNA segments resulted in the hydrophilic groups residing within these molecules, thereby exposing the hydrophobic groups on the surface at 25 °C. This HB interactions also between the PNIPAAm and ssDNA segments weakened significantly at 45 °C due to the predominance of intramolecular HB between the PNIPAAm segments [34], driving the ssDNA segments to the surface and leading to a hydrophilic surface state. While increasing the thickness of the PNIPAAm segment, the SWCA at 25 °C decreased from 78.5° for PN4D to 64.5° for PN8D, while the SWCA at 45 °C increased from 46.9° to 51.7° (Fig. 4b). The SWCAs of PN12D at 25 and 45 °C were 49.8° and 56.3°, respectively. From the SWCAs results, PN12D exhibited minimal thermoresponsive behavior (Fig. 4c). Notably, the thermoresponsive behavior of the copolymer brushes became similar to that of PNIPAAm when grafting of the PNIPAAm segment have been carried out for a longer time than 12 h. The difference in the SWCAs between the hydrophilic (34°) and hydrophobic (62.1°) states reached 28.1° for PN16D (Fig. 4d). For PN20D, the thermally responsive behavior was close to pure PNIPAAm brushes (Fig. 4e). These results indicate that when the thickness was greater than 300 nm, the PNIPAAm segment dominated the surface properties.

### Resistivity change of PNIPAAm-*b*-ssDNA copolymer brushes for complementary

**Fig. 5:**
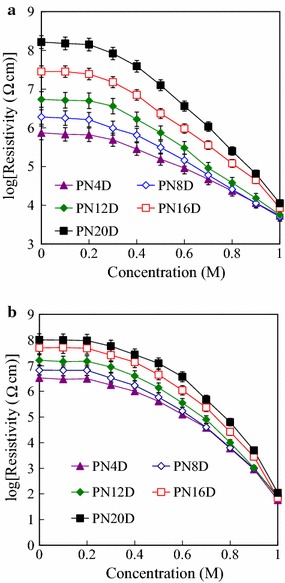
Resistivities of PN4D, PN8D, PN12D, PN16D and PN20D plotted as a function of NaCl concentration at **a** 25 °C and **b** 45 °C, respectively

**Fig. 6:**
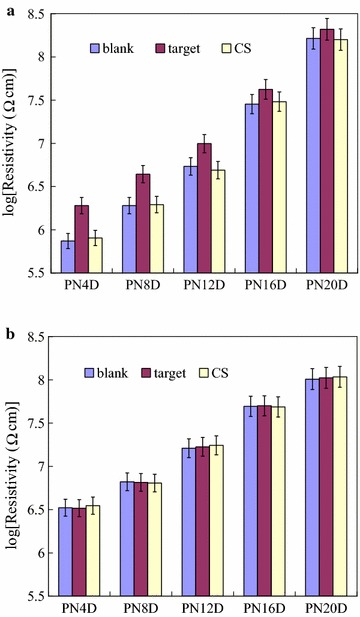
Resistivities of blank PNIPAAm-*b*-ssDNA copolymer brushes (logarithmic scale) and upon hybridization with 0.5 ng/µL target and CS at **a** 25 °C and **b** 45 °C

**Fig. 7:**
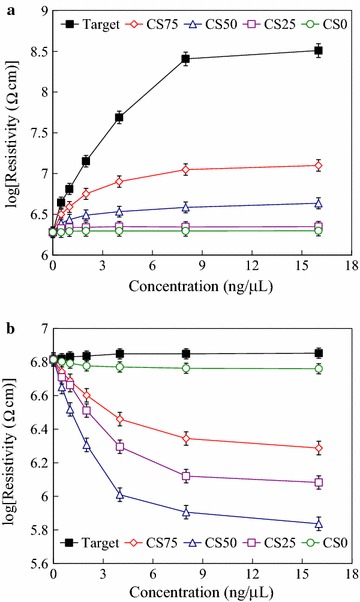
Resistivities of PNIPAAm-*b*-ssDNA copolymer brushes (logarithmic scale) plotted with respect to the hybridization concentration of the target, CS75, CS50, CS25 and CS0 sequences (0.5–16 ng/µL) at **a** 25 °C and **b** 45 °C

**Fig. 8:**
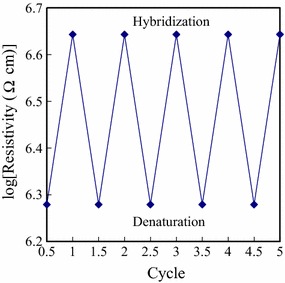
Resistivities of PNIPAAm-*b*-ssDNA copolymer brushes (logarithmic scale) with target hybridization and denaturation for five cycles at 25 °C

As BMHBs can enhance the electron transport in PNIPAAm-*b*-ssDNA copolymer brushes, the determination of the temperature-responsive behavior of the resistivity of the functionalized surface was particularly interested in this study. The obtained linear *I*–*V* curves, which displayed the ohmic behavior, and the slopes of these *I*–*V* curves represents the resistivities of the surfaces at 25 and 45 °C temperatures. Because the salt may affect the resistivities, the thermoresponsiveness of resistivity of the functionalized surfaces of PNIPAAm-*b*-ssDNA copolymer brushes at various salt concentrations of NaCl was investigated. Figure 5 represents the logarithms of the average resistivities of the blank PNIPAAm-*b*-ssDNA copolymer brushes plotted with respect to the NaCl concentrations at 25 and 45 °C, respectively. The resistivities did not change significantly after immersion in the NaCl solution below 0.2 M of concentration at both of 25 and 45 °C. A significant decrease in resistivities for the copolymer was observed upon increasing the NaCl concentration from 0.2 to 1 M at 25 and 45 °C, respectively. The anions and cations of residual salt over 0.2 M within the layers significantly enhance the conductivity, consistent with the results of complexation degree between PNIPAAm segments and DNA. In addition, higher temperature (45 °C) accelerate the motion of the ions over 0.2 M enhanced the conductivity as well. The results suggest that the anions and cations of residual salt predominately determine the resistivity of these layers above 0.2 M NaCl concentration. There was no distinct changes in the logarithms of average resistivities below 0.2 M NaCl concentration exhibited the reliability of the PNIPAAm-*b*-ssDNA copolymer brushes for further DNA detection. The optimal operation window for DNA detection ranges from 0 to 0.2 M. Therefore, the specimens were prepared in the PBS solution at 150 mM NaCl concentration for further experiments. Figure 6 represents the logarithms of the average resistivities of the blank PNIPAAm-*b*-ssDNA copolymer brushes and those upon hybridization at 25 and 45 °C, with the target and CS0 at a concentration of 0.5 ng/µL, as determined from their *I*–*V* curves. Notably, the corresponding *I*–*V* curves for the neat PNIPAAm film was not able to obtain due to non-occurrence of electron transport. Therefore, the resistivity was not to obtain for neat PNIPAAm brushes, which could be considered as an insulator. Upon grafting polymerization with the ssDNA sequence, the electron transport in the films increased significantly as a result of the complexes that formed, which led to a decrease in resistivity. Thus, the neat PNIPAAm film could be changed significantly from an insulator to a semiconductor merely through the grafting polymerization of nucleobases. The resistivities of the PNIPAAm-*b*-ssDNA copolymer brushes increased upon increasing the length of the PNIPAAm segment. The length ratio of the ssDNA to the PNIPAAm segments determined the resistivity of the copolymer brushes, confirming that the increased conductivity of the samples at 25 °C arose from electron transport induced by BMHBs (Fig. 6a). The significant increase in the resistivity of the grafted copolymer layer upon hybridization was observed with the target at 25 °C. The strong complementary HB resulted in phase separation of the PNIPAAm and ssDNA segments, leading to an increase in resistivity. As such, the target sequence could be exploited to eliminate the proton transfer of complexation at 25 °C. Notably, the resistivity could be influenced readily by the humidity or roughness of the surface. The inaccuracy ranges were marked as the indices for recognition of hybridization with the target. A change in resistivity without overlapping the inaccuracy range is defined herein as a distinguishable value. In this work, it was found that the distinguishable resistivities of PN4D, PN8D, and PN12D for hybridization with the target sequence were greater than those of the other samples. Thus, the higher ratio of probe to PNIPAAm segments enhanced the distinguishable resistivity significantly at 25 °C. Furthermore, the usage of sequences CS0 to investigate the distinguishable degrees of complementarity of our surfaces. As compared with hybridization of the target, the resistivities after hybridization with CS0 did not result in a significant decrease of resistivity at 25 °C [35]. The non-hybridization of CS0 at 0.5 ng/µL could be distinguished from the target when using the PNIPAAm-*b*-ssDNA copolymer brushes. At 45 °C, the resistivities of the blank PNIPAAm-*b*-ssDNA copolymer brushes and those upon hybridization with the target and CS0 were similar (Fig. 6b). The results verify that the phase separation of the PNIPAAm and ssDNA segments was not only induced by increasing temperature above the LCST, but also by hybridizing with the target. The PN8D was exploited to investigate the distinguishable resistivity and degrees of complementarity using four kinds of control sequences featuring 0, 25, 50 and 75% of complementarity at 25 and 45 °C, respectively. Figure 7 represents the logarithms of the average resistivities of PN8D plotted with respect to the hybridization concentration of the target, CS75, CS50, CS25 and CS0 sequence (0.5–16 ng/µL) at 25 and 45 °C, respectively. At 25°C, The resistivities of PN8D increased upon increasing the concentrations ng/µL of the sequences to 8. The resistivities reached the plateau, indicating the hybridization saturation of the probe segment, at the concentration more than 8 ng/µL (Fig. 7a). The resistivities did not change significantly upon hybridization with CS0 for various concentrations because of non-hybridization. The DNA segments partially hybridized with the control sequences, CS25, CS50 and CS75, leading to the residual sequences within the copolymer. The mismatched segments of residual sequences facilitated the electron transport (complexation), but matched segments suppressed the electron transport (decomplexation). Even though the resistivities increased with the degree of complementarities of the control sequence for all concentrations. The hybridization of CS25, CS50 and CS75 at 0.5 ng/µL could be distinguished from the target by the resistivities indicating the high distinguishable degrees of complementarity at 25 °C. At 45 °C, the phase separation of copolymer resulted in the similar resistivities upon hybridization with the target and CS0 (Fig. 7b). In addition, hybridization CS25, CS50 and CS75 with the probe segments of the copolymer at various concentrations resulted in the decrease of resistivities. The CS25, CS50 and CS75 did not hybridize completely with the probe of the copolymer mismatched segments facilitated “leakage” of electron transport resulting in the decrease in resistivities. The quantity of residual sequencing within the copolymer decreased with the increase of mismatched segments after cleaning and lyophilizing processes. The resistivities upon hybridization with CS25, CS50 and CS75 followed the order CS75 > CS25 > CS50 at 45 °C. The results suggest that these sequences possessing various degrees of complementarities could be distinguished with PN8D from the target by the resistivities at both of 25 and 45 °C. Figure 8 displays the logarithms of the average resistivities of PN8D with target hybridization and denaturation for five cycles at 25 °C. For the PN8D, this behavior was reversible for at least five cycles, which indicates the excellent recyclability of the PNIPAAm-*b*-ssDNA copolymer brushes. The application of the electrical properties of such copolymers could lead to smart materials capable of detecting specific DNA sequences without labeling.

## Conclusions

In the present study, specific BMHBs between PNIPAAm and nucleobases can generate noncovalently interacting colloidal supramolecular systems within a single chain of a copolymer brush. Such interactions between the PNIPAAm and nucleobase segments results in the changes of the thermoresponsive hydrophobicity and resistivity of the surface. The mode of HB of the PNIPAAm-*b*-ssDNA copolymer brushes can be switched simply by hybridizing with a target DNA sequence with significant changes in the electrical resistivity occurring for the complexed and decomplexed (phase-separated) states. Accordingly, PNIPAAm-*b*-nucleobase brushes in the present study are promising for future theoretical and practical applications.

## Additional file

**Additional file 1: Figure S1.** Thickness of (a) PN4, PN8, PN12, PN16 and PN20, and (b) PN4D, PN8D, PN12D, PN16D and PN20D plotted as a function of temperature.

## Abbreviations

PNIPAAm: poly(N-isopropylacrylamide)
ssDNA: single-stranded DNA
BMHBs: bio-multiple hydrogen bonds
LCST: lower critical solution temperature
