# Supplementary material for: Fabrication of device with poly(N-isopropylacrylamide)-b-ssDNA copolymer brush for resistivity study
Source: J Nanobiotechnology. 2017 Oct 5;15:68. doi: 10.1186/s12951-017-0303-4 (PMC5629771; doi:10.1186/s12951-017-0303-4)
Supplement: Supplementary file 1 — Additional file 1: Figure S1. Thickness of (a) PN4, PN8, PN12, PN16 and PN20, and (b) PN4D, PN8D, PN12D, PN16D and PN20D plotted as a function of temperature. [file 12951_2017_303_MOESM1_ESM.doc]

**Supplemental information**

(a)

(b)

Figure S1: Thickness of (a) PN4, PN8, PN12, PN16 and PN20, and (b) PN4D, PN8D, PN12D, PN16D and PN20D plotted as a function of temperature.
